# The integration of lipid-sensing and anti-inflammatory effects: how the PPARs play a role in metabolic balance

**DOI:** 10.1186/1478-1336-5-1

**Published:** 2007-05-25

**Authors:** Alistair VW Nunn, Jimmy Bell, Philip Barter

**Affiliations:** 1 Molecular Imaging Group, Medical Research Council Clinical Sciences Centre, Imperial College, Hammersmith Campus, London W12 0HS, UK; 2 The Heart Research Institute, Camperdown, Sydney, NSW 2050, Australia

## Abstract

The peroxisomal proliferating-activated receptors (PPARs) are lipid-sensing transcription factors that have a role in embryonic development, but are primarily known for modulating energy metabolism, lipid storage, and transport, as well as inflammation and wound healing. Currently, there is no consensus as to the overall combined function of PPARs and why they evolved. We hypothesize that the PPARs had to evolve to integrate lipid storage and burning with the ability to reduce oxidative stress, as energy storage is essential for survival and resistance to injury/infection, but the latter increases oxidative stress and may reduce median survival (functional longevity). In a sense, PPARs may be an evolutionary solution to something we call the 'hypoxia-lipid' conundrum, where the ability to store and burn fat is essential for survival, but is a 'double-edged sword', as fats are potentially highly toxic. Ways in which PPARs may reduce oxidative stress involve modulation of mitochondrial uncoupling protein (UCP) expression (thus reducing reactive oxygen species, ROS), optimising forkhead box class O factor (FOXO) activity (by improving whole body insulin sensitivity) and suppressing NFkB (at the transcriptional level). In light of this, we therefore postulate that inflammation-induced PPAR downregulation engenders many of the signs and symptoms of the metabolic syndrome, which shares many features with the acute phase response (APR) and is the opposite of the phenotype associated with calorie restriction and high FOXO activity. In genetically susceptible individuals (displaying the naturally mildly insulin resistant 'thrifty genotype'), suboptimal PPAR activity may follow an exaggerated but natural adipose tissue-related inflammatory signal induced by excessive calories and reduced physical activity, which normally couples energy storage with the ability to mount an immune response. This is further worsened when pancreatic decompensation occurs, resulting in gluco-oxidative stress and lipotoxicity, increased inflammatory insulin resistance and oxidative stress. Reactivating PPARs may restore a metabolic balance and help to adapt the phenotype to a modern lifestyle.

## Background

Peroxisomal proliferating-activated receptors (PPARs) were discovered in 1990 with the cloning of a murine orphan receptor that was activated by peroxisomal proliferating compounds (such as the fibrates), hence their name [1]. They probably arose during metazoan evolution and at least three isoforms have been identified, α, γ and δ (also referred to as PPAR β), each encoded by a different gene [2]. They are ligand-activated transcription factors that act as lipid sensors and work as dimers with the retinoid X receptor (RXR), detecting a broad range of molecules (including inflammatory lipid mediators) and modulate the activity of genes involved in energy regulation and inflammatory processes, including wound healing, as well as reproduction [3-6]. They are also important in embryonic development, but only PPAR γ knockout is lethal – although placental rescue results in a phenotype with no body fat, which confirms its pivotal role in adipogenesis [2]. They therefore appear to be involved in many (apparently disparate) metabolic processes, which therefore raises a question, why did they evolve and what is their overall function?

We believe that PPARs may be an evolutionary solution to something we call the 'hypoxia-lipid' conundrum, where the ability to store and burn fat is essential for survival, but is a 'double-edged sword', as fats are potentially highly toxic. For instance, hypoxia results in the increased production of mitochondrial ROS, which can result in lipid peroxidation that is not only potentially damaging, but also a strong inflammatory signal, activating nuclear factor kappa-beta (NFkB) [7]. Thus, a group of transcription factors that integrate resistance to oxidative stress (inflammation, thus modulation of NFkB), with the ability to detect and orchestrate the storage and metabolism of lipids, while sparing glucose (which can be burnt anaerobically), was inevitable. Over time, this function engendered increasing functional longevity, and ultimately, as they evolved (they have been one of the fastest evolving group of nuclear receptors) [8], this may have enabled the evolution of longer lifespans for some species.

Key in this, we believe, may be their ability to modulate uncoupling proteins activity (UCPs), so reducing mitochondrial reactive oxygen species production (ROS), as well as their ability to induce insulin sensitisation – so optimising forkhead box class O factor (FOXO) activity by reducing insulin basal levels and therefore insulin 'drive': FOXO are a small subfamily of transcription factors key in stress resistance and calorie restriction-induced longevity, whose function is suppressed by high insulin/IGF-1 activity (reviewed by Morris BJ, 2005) [9]. Increased expression/activity of FOXO results in increased activity of peroxisome proliferator-activated receptor gamma coactivator-1α (PGC-1α), which also plays a key role in longevity and the calorie restriction phenotype, in particular, it increases the expression of PPAR α [10]: 19% of the genes that are regulated during calorie restriction are modulated by PPAR α-including suppression of acute phase response (APR) genes [11]. These nuclear factors are also upregulated by exercise [12], which is known to improve median survival.

Overall, the ability of all the PPARs to reduce lipotoxicity and suppress inflammation would strongly suggest that they would all tend to reduce the need for basal insulin by encouraging insulin sensitivity. This indicates that as an ancient group of nuclear factors, which are essential for fat storage and metabolism, they are also key in suppressing oxidative stress: the ability to store fat and resist oxidative stress are both generally associated with improved survival and increased species lifespan [13,14]. We suggest that the phenotype associated with calorie restriction is thus the opposite of that seen with the metabolic syndrome and the balance between the two may be determined, to a large degree, by PPAR activity.

## The transcriptional triad of survival: PPAR-FOXO-NFkB

**Figure 1:**
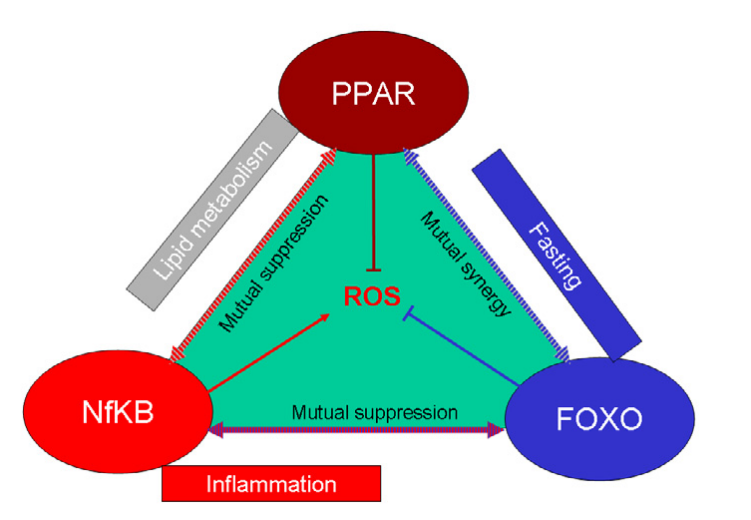
**Transcriptional triad of survival**. PPARs promote mitochondrial proton gradient uncoupling, reduce ROS and increase heat generation, while ensuring safe lipid storage and burning (reducing lipotoxicity), safe carbohydrate storage and reducing need for insulin. They also suppress inflammation. NFkB promotes resistance to infections and aids healing, but suppresses incentive salience and increases thermogenesis – it can be said to have general anorexic actions. Also promotes ROS production, both as a signal and as a defence. Response amplified by increasing fat stores. Promotes inflammation. FOXO promotes resistance to oxidative stress, enhances DNA repair, suppresses proliferation, and encourages incentive salience and survival in low food situations – it is thought to be generally orexigenic. Can oppose inflammation.

Although each PPAR isoform is expressed in almost every tissue, they are expressed in a tissue- and time-specific manner in response to food, as well as to exercise and cold. PPAR α is very active during fasting and is predominantly found in the liver, while PPAR γ is active during feeding and is predominantly found in adipose tissue, where its main role appears to enable the deposition of fat: PPAR δ is ubiquitous, with its highest expression in the gut, but is now thought to be extremely important in exercise-induced switch to oxidative (type 1) myofibres, as well as in thermogenesis [13,15,16]. Overall, PPAR γ is thought to be essential for adipogenesis (and thus storage), whereas PPARs α/δ are more involved in fatty acid catabolism. Importantly, their expression and activity is intimately related to other transcription factors/co-factors, in particular, FOXO, PGC-1 and NFkB: figure 1 is simplified explanation of how these transcription factors might interact – the 'transcriptional triad'.

### The calorie-restriction and longevity connection

FOXO is an important group of transcription factors that integrate energy metabolism with resistance to oxidative stress, as well as regulating cell cycle and DNA repair; for example they increase SOD (superoxide dismutase) and also modulate hunger [17]. PGC-1 was first discovered as a cold-inducible co-activator that regulates adaptive thermogenesis, and along with PPARs, induces mitochondrial uncoupling via the UCP-1, so generating warmth [18]. PGC-1 is also essential in controlling hepatic gluconeogenesis, which occurs in response to famine; it is thought to be a "master switch" that controls the change from carbohydrate- to fat-based metabolism, which includes a change from type II to type I muscle fibre use and increased mitochondrial biogenesis [19]. To be activated, PGC-1 requires interaction with FOXO1 [20]. Once activated, PGC-1 cooperates with PPAR α to activate genes encoding mitochondrial enzymes involved in fatty acid oxidation [21]: PPAR α is also of major importance in calorie restriction [11]. FOXO1 can inhibit PPAR γ activity in adipose cells and vice versa [22], but it can enhance PPAR α activation of lipoprotein lipase in muscle [23]. This would support the flow of fatty acids from adipose tissue during fasting to energy requiring tissues (and thus shrinkage of the adipose store), but the flow of fats into adipose tissue during feeding.

PPAR activity decreases with age, a process that can be slowed by calorie restriction [24], while aging is associated with increased constitutive activity of NFkB [25]. Indeed, it has been suggested that PPARs may play a role in modulating the 'molecular inflammatory process of ageing' [26], and may be important in suppressing the ageing-associated increase in NFκB activity [27]. Certainly, calorie restriction has been shown to result in a generalised increase in PPAR activity, which is associated with increased adiponectin [28]; adiponectin can also suppress NFkB activity [29]. This would be supported by the well described observation that pharmaceutical activation of PPARs α & γ is broadly beneficial, reversing many aspects of the metabolic syndrome; the same is now thought to be true for PPAR δ [30]. At the transcriptional level, NFκB and FOXO do appear to have mutually exclusive activity, as IkB (inhibitor of NFkB) kinase (IKK), can result in the activation of NFkB by inhibiting IkB, but the direct inhibition of FOXO, which maybe be important in cancer [31]. In addition, NFkB and PPARs can also trans-repress each others activity [32-35]. Hence, there is both anecdotal and transcriptional evidence that PPAR activity is associated with a longer-lived phenotype.

### Why adipose tissue is inflammatory, and why PPARs are anti-inflammatory

**Figure 2:**
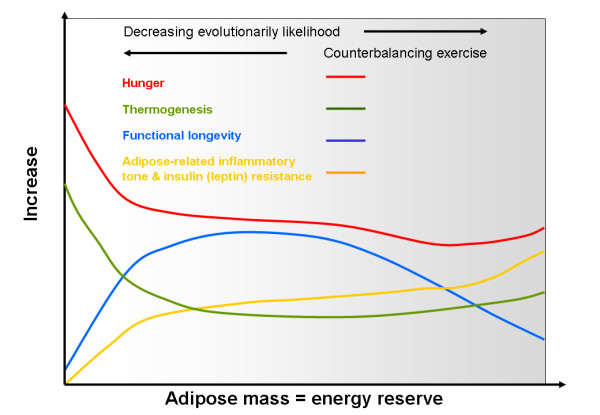
**Relationship between energy storage, inflammation, thermogenesis, hunger and survival**. As fat mass increases, it sends out a proportionally bigger inflammatory signal that also induces insulin resistance: this might compensate for the normal inflammatory suppression of orexia. Thus, it is a normal survival response selected for by evolution, as both storage of energy, and the ability to mount a strong immune response, were strong survival traits. It is likely that during ancient times there was never such a thing as a 'free lunch'; before the advent of civilisation it was unlikely that there were extended periods (e.g. beyond a year) when food was plentiful and there was little need to move to get it.

One of the more interesting aspects of the PPARs is that they seem to integrate inflammation and energy metabolism. It is now becoming apparent that adipose tissue is metabolically very active and increasing adipose mass is associated with increasing inflammatory tone. It is now thought that this may be an evolutionarily technique to enhance survival in relation to famine and immunity/inflammation, which are both highly energy dependent: one key signal for this may be leptin [36]. This would explain why excessive obesity is generally associated with sub-clinical inflammation, and why there is generally an evolutionarily-driven imbalance between orexigenic (stronger) and anorexic (weaker) signals, leading to high feed-efficiency and a propensity to store fat [37-39]. However, it would also be an evolutionary trade-off, as a food-rich environment, with little need for physical activity, might continually increase fat mass and lead to increasing oxidative stress (and thus, production of ROS) and a shortened lifespan (figure 2) – this could be an example of antagonistic pleiotropy. Certainly, obese adipose tissue can attract macrophages, resulting in a heightened inflammatory response – which can be reversed by weight loss [40]. Thus the finding that PPAR γ activation can induce apoptosis of macrophages found in adipose tissue [41], might suggest that not only does PPAR γ ensure fat storage, but that it might also suppress the adipose-related inflammation signal.

This therefore supports the 'transcriptional triad of survival' paradigm (figure 1). FOXO, which is mainly active during fasting/famine, maintains resistance to oxidative stress and improves long-term survival: NFkB, which is highly important in resistance to injury/infection, engenders oxidative stress as a survival strategy. However, PPARs, are essential to ensure that energy-related oxidative stress is kept to a minimum, either during storage, or during metabolism (such as exercise, fasting or infection).

## The hypoxia-lipid conundrum

The main problem with lipids is that they require oxygen to be burnt as fuel. However, the only way to do this is via mitochondria, which are also one of the prime cellular sources of ROS; ROS production is increased when levels of the ultimate electron acceptor, oxygen, is decreased. Thus, there is the potential for lipid peroxidation and lipotoxicity. We suggest that the ability to utilize lipids as fuel has to have co-evolved with the ability to suppress oxidative stress: PPARs may well be a very important part of the solution. In contrast, carbohydrate metabolism has become associated with inflammation.

### Fat versus glucose: PPARs and energy source switching

Carbohydrate is an essential fuel source for the CNS and immune system, but excess carbohydrate is stored as energy-dense fat, a process that requires mitochondria and the participation of PPARs. The ability to store and metabolize fat is an important survival mechanism as it provides energy when food is scarce; it is far more energy dense than glycogen. During fasting, glycerol can be released from adipose stores, which can then be used by the liver for gluconeogenesis – thus providing carbohydrate to for the CNS. Studies indicate that the evolution of a longer lifespan has been associated with the development of a higher body mass and an increased percentage of body fat [13,42]. Thus, fat storage is a positive survival trait and the adipogenic function of PPAR γ makes it tightly linked into long-term survival.

Fats, in addition to being stored, must also be readily made available for oxidation. PPAR α and PPAR δ are active in muscle and ensure entry of fats into the β-oxidation pathway, as illustrated by their upregulation during endurance exercise [43,44] or thermogenesis [18]. However, although an increased lifespan is dependent on the ability of an organism to store and utilize energy-dense fat, there is an inherent problem in using fats as an energy substrate; they are highly susceptible to ROS damage, which is especially true for unsaturated fatty acids (because of readily oxidisable double-bonds) [45].

Under normal conditions, oxygen utilization is closely coupled to energy production and expenditure. However, in hypoxic circumstances, for example during localised biological stress induced by injury/infection (where blood flow is compromised), the production of mitochondrial ROS is significantly increased due to a reduction of oxygen as an electron acceptor. This may have become part of an ancient mitochondrial-based oxygen sensing/signalling mechanism, which involves the hypoxia-induced factor-1 (HIF-1) transcription factor [46]. It is also likely that ROS signalling plays a key role in the activation of NFkB [47] and thus, in inflammation [48]. During hypoxia, carbohydrate becomes the more important fuel source as it is more oxygen-efficient and if necessary, can undergo anaerobic respiration (fats cannot be burnt without oxygen and mitochondria). HIF-1 can inhibit both PPAR α and PPAR γ expression [49,50], but may require NFκB for full activity [51,52]. Certainly, activation of NFkB in cardiac muscle can suppress the transcriptional activity of both PPAR α & δ, suggesting a switch to carbohydrate metabolism [33].

As indicated above, HIF-1 downregulates PPAR activity in a hypoxic environment causing a switch to carbohydrate burning. However, the increased ROS production may well be negatively regulated PPARs, as oxidised lipids are potent ligands for the PPARs. Thus, although hypoxia can trigger ROS production and the ability to switch off beta-oxidation, the process is self-regulated by the anti-inflammation actions of the PPARs – thus ensuring minimal duration of oxidative stress. Certainly, PPAR α is known to suppress the dehydrogenase pyruvate complex (PDC), by upregulation of pyruvate dehyrogenase kinase 4 (PDK4) during starvation [53], which would reinforce its role in energy switching and carbohydrate sparing.

### PPARs and uncoupling proteins: managing ROS and lipids

PPAR activation can increase the expression of mitochondrial UCPs [54-57] – a family of homologues that can 'uncouple' the proton gradient in the mitochondria and so reduce ROS [58]. The activity of UCPs is increased during starvation and by a ketogenic diet [59,60]. They can be directly activated by fatty acids [61], with unsaturated fatty acids being particularly effective [62-64].

Mammals have at least five UCP homologues: UCPs are also found in plants and fungi, and belong to an ancient superfamily of mitochondrial metabolite carriers [65]. It was originally thought that one of their prime functions was to uncouple the mitochondrial deltapsi gradient (so reducing ROS production), and certainly for UCP-1 (which was the first to be discovered) this is true – as it plays a critical role in thermogenesis [18]. However, a precise role for the other UCPs is still being defined, as it is now thought that their primary function maybe as anionic transporters – although a secondary protonophore function may still be very important. For instance, UCP-3 is thought to transport fatty acids out of the mitochondrial matrix (in exchange for a proton equivalent) and is predominantly found in muscle and adipose tissue and is induced by fasting (when lipid levels rise), and thus may play a role in preventing lipotoxicity; this would certainly be supported by the observation that it is decreased in the muscles of T2D patients, and its levels can be restored by PPAR γ activation [66].

Hence, fatty acids may potentially reduce ROS production by directly stimulating UCPs, and the potency of unsaturated fats may reflect their susceptibility to oxidation. Importantly, these same fatty acids would also activate PPARs and increase the expression of UCP (s). Thus, we hypothesize that PPARs may have a vital role to play in reducing hypoxia-related lipid damage through their induction of UCPs and, in so doing, improve functional longevity by suppressing ROS production (and reducing the potential for lipotoxicity); this would be enhanced by their well-described ability to increase the activity of other antioxidant enzymes, such as superoxide dismutaste (SOD) or catalase [67-69].

## Shifting the phenotype; reducing the need for insulin

The ability of PPAR γ to improve glucose dispersal is well described, and is now being described for PPAR δ [70] and PPAR α (although there have been some conflicting results) [71,72]. One of the main ways they do this is to channel fatty acids to where they are needed; this prevents the build up of excessive intramyocellular lipid, which is thought to be one of the major causes of insulin resistance in obesity [73]. In addition, it is also becoming clear that an increase in ROS can also cause insulin resistance [74]. This would support the observation that increasing free fatty acid (FFA) concentrations can induce NFkB activity (and ROS) [75], and in the liver, this may partly explain hepatic insulin resistance [76]. However, it has been long known that ROS is involved in insulin (and that of other growth factors) signalling, possibly through NADPH oxidase (Nox) production of H_2_O_2 _(reviewed by Goldstein, Mahadev 2005) [77]. This would imply that not only is control of intracellular redox vital, but that excessive insulin signalling, (apart from suppressing FOXO), may also contribute to increased cellular oxidative stress.

It has been proposed for some time that 'thrifty' genes would have given our ancestors a survival edge in harder times by enabling rapid storage of fat, but in times of plenty, may have resulted in increased levels of diabetes [78]; key to this insulin resistance may be the role of lipids. In contrast, in times of plenty selective pressure may have resulted in 'unthrifty' genes, which would be associated with insulin sensitivity. For instance, the PPAR γ ala allele, which is diabetes protective and probably arose between 32,000 to 58,000 years ago [79]. Thus, both insulin resistance and sensitivity can be potential survival traits – so what is the role of the PPARs in determining this balance?

### Keeping FOXO active: PPARs modulate insulin 'drive'

The facility of a rapid food-induced insulin response and the ability to store food efficiently after starvation, while retaining a degree of insulin resistance post-prandially, may be example of a "thrifty adaptation" to spare glucose for the CNS and provide energy for muscle during times of hardship – both for movement and thermogenesis [80]. Certainly, muscle insulin resistance combined with adipose insulin sensitivity may comprise a 'fat catch-up' paradigm by ensuring fatty acid channelling to adipose tissue and decreased muscle thermogenesis [81]. In addition, the recent discovery of a hormone associated with longevity called 'klotho' that can induce insulin resistance and upregulate FOXO [82] is therefore significant, as is supports the observation that the right degree of insulin resistance may aid long-term survival. For instance, if normal human subjects are starved for 48 hours, they become insulin resistance, which is thought to be a natural response to maintain glucose levels and is related to decreased glucose dispersal by down-regulation of muscle PDC [83]. This insulin resistance is probably related to increased intramyocellular fat, as during starvation, FFA levels rise [84]. Similarly, 60 hour starvation of patients with T2D or obesity can result in increased insulin resistance, but only in those who were relatively insulin sensitive to begin with; in some highly insulin resistant patients, starvation improved sensitivity [85]. Indeed, it has been observed for many years that crash dieting can actually induce severe insulin resistance and T2D is some obese patients [86].

FOXO, which is one of the most important transcription factors in improving functional longevity during fasting, is negatively regulated by insulin [9]. We suggest that a critical function of the PPARs is to reduce insulin "drive" (via appropriate tissue insulin sensitisation) and thereby increase functional longevity by preventing the insulin-mediated downregulation of FOXO. This process is also extended directly to insulin production, as PPARs are involved in controlling glucose-stimulated insulin release, a process that is modulated by fatty acids and may involve UCPs: increased PPAR α activity is associated with down regulation of insulin production during fasting, while PPAR γ islet over-expression can also suppress insulin release [87,88]. Interestingly, saturated fat is far more insulinotropic than unsaturated fat [89], which might suggest that PPARs are more effective at reducing insulin production in response to unsaturated fats. This is in keeping with the susceptibility of unsaturated fats to oxidative damage. In contrast, saturated fat is less effective than unsaturated fat at stimulating the incretin, glucagon-like peptide-1 (GLP-1), from the gut [90]. The biological activities of GLP-1 include stimulation of glucose-dependent insulin secretion and insulin biosynthesis, inhibition of glucagon secretion and gastric emptying, and inhibition of food intake. This may suggest an evolved bias towards unsaturated dietary fat intake from the gut, but an internal system to react to nascent saturated fat produced from glucose (or fructose): i.e. we are far more able to tolerate ingestion of unsaturated fat, compared to saturated fat – but the system is designed to recognise and deal with *de novo *saturated fat generated from carbohydrate. Human data suggest that rosiglitazone can activate desaturases, so reducing levels of saturated fat in the system [91], which would further indicate that reduction of excess saturated fat is a biological imperative.

We propose that at it simplest, muscle insulin sensitivity may result in increased thermogenesis through futile cycling and thus, would be associated with an 'unthrifty' genotype. Key in either the thrifty, or unthrifty genotypes (as indicated by the PPAR ala/pro mutation), would be the role of the PPARs: increased adipose PPAR γ activity would result in better fat storage (adipose insulin sensitivity), whereas an improved ability to burn fat in muscle (PPAR α/δ) might be associated with better muscle insulin sensitivity and less efficient feed efficiency (but a better tolerance to cold). Hence, by modulating tissue-specific fatty acid metabolism and storage, PPARs are able to maximise FOXO activity and thus optimise resistance to oxidative stress by reducing the need for insulin. One obvious exception to this is the mutually suppressive effects of PPAR γ and FOXO in adipose tissue [22]; increased PPAR γ activity would act to store fatty acids, while still maintaining an anti-inflammatory effect (reduce oxidative stress) by suppression of NFkB. Certainly, basal NFκB activity increases during adipocyte differentiation [92]. This would suggest a possible adipose-inflammatory paradigm, whereby increased NFkB activity could conceivably suppress both FOXO and PPAR γ, resulting in 'inflammatory' lipolysis. During starvation, FOXO would be expected to suppress both NFkB and PPAR γ and result in 'starvation' lipolysis. However, in obesity, this natural suppression of inflammation is lost due to the high adipose-related inflammatory signal, which suppresses both PPAR γ and FOXO: this could lead to the metabolic syndrome.

### The metabolic syndrome; PPARs keep the acute phase response in check

It has been suggested that in addition to the 'thrifty' genotype, another adaptation may also be needed to develop the metabolic syndrome, and that is a 'high cytokine responder' genotype, with an improved ability to resist injury (i.e. a stronger inflammatory response) [93]. It has been known for many years that injury can result in profound insulin resistance and is associated with the APR, which is a systemic inflammatory injury response to protect the host (being both haemostatic and anti-microbial) characterised by the hepatic production of acute phase proteins (e.g. c-reactive peptide, CRP) and glucose, increased cytokine production and turnover of protein, glycerol free and fatty acids, and has been called the 'hypermetabolic response' [94,95]. This 'hypermetabolic' (catabolic) state can be mimicked by injection of the stress hormones cortisol, glucagon and ephedrine in human volunteers [96]. However, this 'hypermetabolic' state is usually associated with increased thermogenesis (pyrexia) and is anorexic, and probably involves inflammatory-mediated modulation of appetite systems, such as the melanocortin pathway [97]; this is clearly not the case in the metabolic syndrome. Interestingly, leptin is known to mediate the effects of lipopolysaccharide (LPS) induced anorexia and fever [98], but central leptin (and insulin) resistance is a common finding in obesity and could be related to leptin itself via effects on phosphatidylinositol 3-kinase (PI3K) and phosphodiesterase 3B (PDE3B) activities and reduction in cyclic AMP (cAMP) [99] and/or the pro-inflammatory effects of a high fat diet [100]. This might also represent a another thrifty adaptation to ensure a high state of 'inflammatory readiness', but conservation of energy stores.

It was suggested by Pickup and colleagues in 1997 that 'syndrome X' (now called the metabolic syndrome) was in fact a disease caused by the chronic activation of the innate immune system and contributed to the hypertriglyceridaemia, low HDL cholesterol, hypertension, glucose intolerance, insulin resistance and accelerated atherosclerosis of NIDDM [101]. This hypothesis for the development of T2D (and the metabolic syndrome) was further supported by data from the Athersclerosis Risk in Communities study (ARIC) [102]. Importantly, the APR and inflammation can result in increased insulin output, which can in turn suppress the APR – so providing a possible negative feedback mechanism [103]. Interestingly, IL-6, a potent inflammatory cytokine-inducer of the APR produced by adipose tissue, is significantly associated with insulin resistance and insulin levels in men: this relationship may further indicate a 'thrifty' adaptation, which both enhances resistance to infection and ability to store energy [104]. Mutations in the IL-6 gene are associated with increased risk of diabetes [105].

The APR response has now been shown to down-regulate PPAR activity in most tissues, including adipocytes [106] – which is to be expected, as PPARs are generally anti-inflammatory and improve insulin sensitivity. Indeed, they have been described as negative acute phase proteins [107]. At the site of injury (due to hypoxia), HIF-1 (and NFkB) may suppress PPAR activity directly. Away from the site of injury, pro-inflammatory mediators such as angiotensin II can mediate many of the effects of the APR via activation of NFkB and thereby also inhibit PPAR activity [108,109].

**Figure 3:**
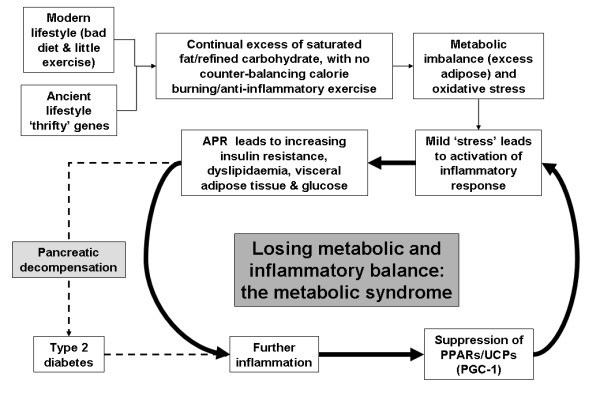
**A modern imbalance: an out of control 'thrifty' response**. Proposal as to what happens when an ancient hunter-gatherer genotype meets a modern lifestyle. Thrifty response and expanding adipose tissue eventually leads to an over-whelming inflammatory signal.

We suggest that at some point in the clinical evolution of the metabolic syndrome and T2D a 'tipping point' is reached, resulting in an inflammatory-driven downregulation of PPARs (figure 3). All of this would strongly indicate that the metabolic syndrome is an exaggerated thrifty response, characterised by insulin resistance, which not only induces a propensity to store fat, but results in a fat-mass related activation of the APR (and resistance to its normal suppression by insulin). However, unlike the well described hypermetabolic injury response, appetite is maintained and thermogenesis suppressed (see below for possible explanation).

## Evolutionary function of the PPARs: putting the brake on oxidative stress

**Figure 4:**
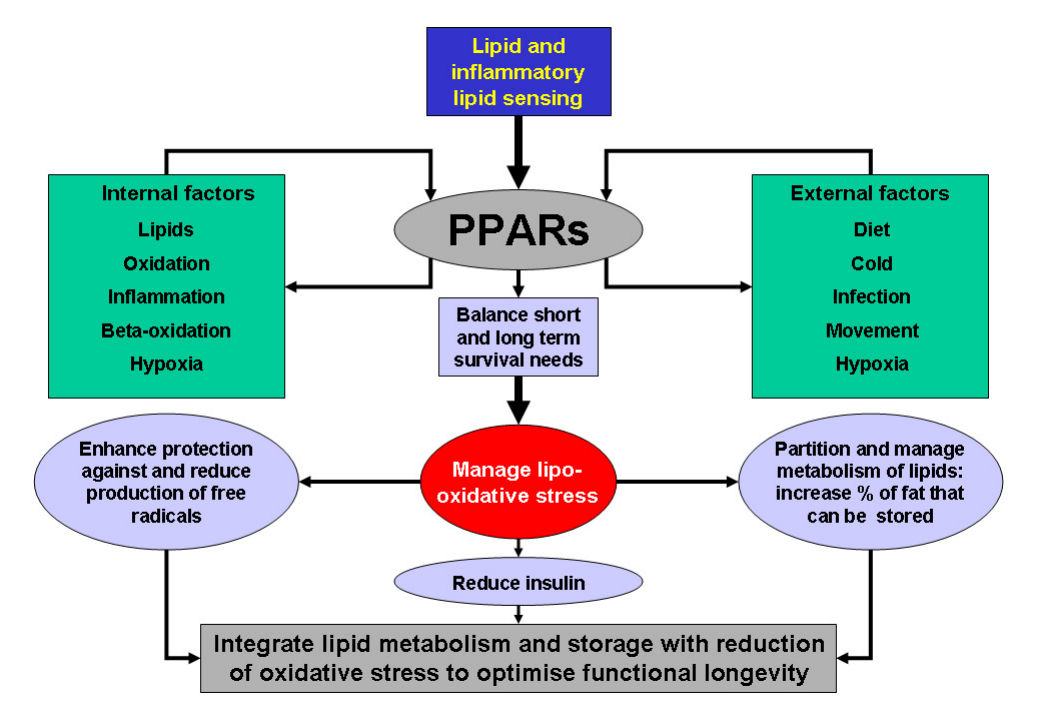
**A possible function for the PPARs**. A summary proposing the overall function of the PPARs (peroxisomal-proliferating activated receptors).

In summary, we postulate that as transcriptional factors, PPARs have evolved to improve functional longevity by integrating lipid storage and burning with both reduction of insulin levels and suppression/resolution of inflammation and thus reduction of ROS and oxidative stress (figure 4). This critical role is summarised by the 'transcriptional triad' (figure 1). Key in this is the ability of PPARs to overcome the 'hypoxia-lipid conundrum', so preventing hypoxia-driven lipid damage and excessive activation of the APR: insulin resistance is induced by increased ROS, inflammatory mediators and deposition of intramyocellular lipids – all things normally suppressed by the PPARs. By reducing the need for insulin, they can optimise FOXO, which upregulates many genes involved in resistance to oxidative stress. Although PPARs have been mostly shown to decrease oxidative stress, there are reports of PPAR γ ligands increasing ROS – especially in cancer cells, which leads to apoptosis and is associated with mitochondrial dysfunction [110-112]. Given the reliance of many cancer cells on glycolysis (the 'Warburg' effect) and the fact that cancer invokes many inflammatory pathways [113], this could be viewed as another mechanism to ensure functional longevity.

The key to understanding the PPARs is their role as lipid sensors in transcriptional control during starvation, feeding and inflammation: fat is essential for long-term survival. During fasting/starvation, muscle insulin resistance is clearly a thrifty response and is associated with fat deposition, and enables glucose sparing, while in the liver, it encourages gluconeogenesis: at its simplest fatty acids and glycerol flow out of adipose tissue to supply energy. During fasting, many tissues start to burn fats – a process that requires PPAR α and δ. In contrast, during feeding, it is necessary to store and/or replenish energy – either as glycogen, or as lipid in adipose tissue: a degree of muscle insulin resistance, and adipose tissue insulin sensitivity, will channel lipid to the correct store. Key in this is PPAR γ. However, this balance is determined by muscle mass: more muscle means higher glucose dispersal, but muscle is also metabolically active and an important site of thermogenesis, and therefore, energy loss – which, we suggest, may be partly determined by mass action and futile cycling (the muscle 'metabolite door' is more open due to insulin sensitivity). Certainly, PPAR δ (and probably α) are important the utilisation of lipid energy muscle. During injury/inflammation, energy is required for the immune system, so insulin resistance is increased and lipids flow out of adipose tissue – which is not to dissimilar to the fasting response. However, unlike the fasting response, the insulin resistance is likely to be largely cytokine induced, plus, it is usually associated with pyrexia (increased futile cycling & thermogenesis) and anorexia – it is a hypermetabolic response. Furthermore, unlike fasting, inflammation is associated with increased oxidative stress. It is also associated with suppression of PPAR activity – however, PPARs would act as negative regulators to modulate the response and improve insulin action.

The transcriptional adaptive response to fasting involves upregulation of FOXO, PGC-1α and PPAR α – all of which enhance resistance to oxidative stress and the ability to burn fat, while ensuring glucose sparing. Thus, insulin resistance is essential for survival, but too much can result in a feed-forward "feed-inflammatory" response: the 'thrifty' response out of control – as it also suppresses the hypothalamic satiety effects of insulin and leptin. At the transcriptional level, FOXO activity appears to be orexigenic in the hypothalamus [114] – insulin (and inflammatory mediators) would suppress its activity. Hence, PPARs, by suppressing oxidative stress and improving insulin action, can prevent this from happening.

### The out of control 'thrifty' response: new lifestyle, old genes – a modern PPAR imbalance

It is now well accepted that "modern epidemics" such as obesity, the metabolic syndrome, and type 2 diabetes reflect that the modern human is simply "drowning in a sea of saturated fats". It is generally thought that this is due to a ancient genotype being exposed to a modern environment. Thus, if we accept that we are simply "Stone Agers in the fast lane" [115], we need to rebalance our systems, either artificially or naturally, to protect us against the ravages of modern living and to slow the aging process. For instance, evidence suggests that dietary manipulation to increase the polyunsaturated/saturated fat ratio can be advantageous [116-118], and the benefits of physical activity are well known, including the ability to delay and prevent type 2 diabetes [119]. One might argue, however, that sections of the population exhibiting the "thrifty genotype" may be especially at risk and need to dramatically change their diet and lifestyle in order to optimize their functional longevity. In many respects, the long-lived calorie-restriction phenotype is the complete opposite of the shorter-lived metabolic syndrome phenotype. Evidence suggests that our ancestors did not often live much beyond the age of 40, so an ability to store fat quickly, which accentuated the potency of the innate immune – was never really a problem in the long term: it is probably an example of 'antagonistic pleiotropy', which gave a survival advantage while young, ensuring reproductive success.

**Figure 5:**
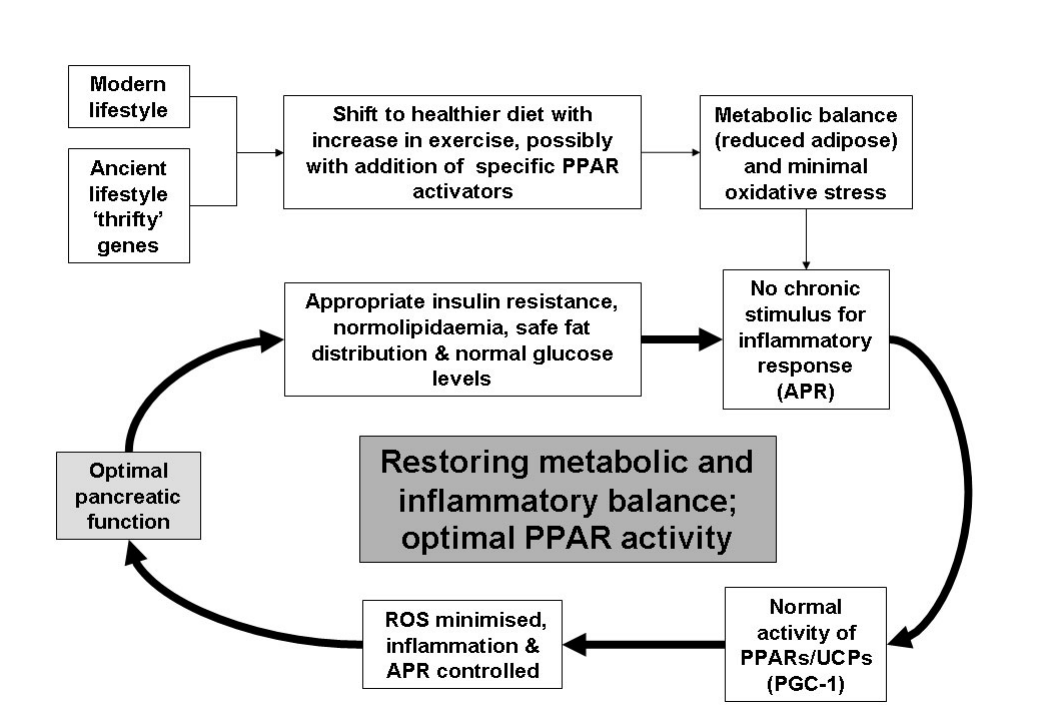
**Restoring metabolic balance**. Lifestyle changes and therapeutic interventions in redressing the imbalance leading to restoration of metabolic and inflammatory balance. Sustained weight loss critical for optimum results.

Modulation of PPARs appears to occur in the APR, which occurs as part of an injury-related response. During this reaction to injury, it may be necessary to downregulate PPARs, as they have many effects that are counterproductive to tissue healing (e.g., they reduce insulin, resistance, suppress expression of inflammatory cytokines and are anti-proliferative). Many of the pathways that are thought to achieve this downregulation are involved in allowing the metabolism of fats. Significantly, many of the signs and symptoms of the APR are shared by the metabolic syndrome. Therefore, it is not untoward to speculate that the downregulation of PPARs may well be at the root of the metabolic syndrome. The cause of this downregulation may well be the modern lifestyle, leading to an out of control 'thrifty response'. Modulating PPARs may be one way to help prevent this. In this respect, it may be possible to "rebalance" our systems through changes in diet, lifestyle, and, possibly, therapeutic intervention (figure 5) – the PPARs may therefore play a very important role in 'metabolic balance'. The benefits of PPAR activation/modulation are clear from trials where PPARs have been activated by pharmacological and natural ligands [120,121]. However, the increased mortality of some patients with compromised cardiac function in trials with the newer dual PPAR agonists does suggest that modulation of PPAR function needs to be handled carefully in some populations [122], as enhancing their activity may counter-act an ancient injury survival system.

## Competing interests

We received financial support from AstraZeneca for writing and editorial  assistance from Chris Langford, PhD, and Lyndsey Wood of PAREXEL MMS Europe Ltd.

Alistair Nunn is a consultant for GW pharmaceuticals.    

Jimmy Bell has no financial relationships to disclose.      

Philip Barter has financial relationships with the manufacturers of  healthcare products that include the following: employment, no conflict of  interest; consultancy, AstraZeneca, Pfizer, and Sanofi-Aventis; honoraria  received, AstraZeneca, Fournier, Merck, Pfizer, and Sanofi-Aventis; research  support or collaboration, Pfizer; participation in sponsored clinical trial,  AstraZeneca, Fournier, and Pfizer; lecture/speaker's bureau/other fees,  AstraZeneca, Fournier, Merck, Pfizer, and Sanofi-Aventis; shareholder, nil;  patent holder in the field, nil.
